# Termites have wider thermal limits to cope with environmental conditions in savannas

**DOI:** 10.1111/1365-2656.13673

**Published:** 2022-02-24

**Authors:** Joel S. Woon, David Atkinson, Stephen Adu‐Bredu, Paul Eggleton, Catherine L. Parr

**Affiliations:** ^1^ School of Environmental Sciences University of Liverpool Liverpool UK; ^2^ Department of Life Sciences Natural History Museum London UK; ^3^ Department of Evolution, Ecology and Behaviour University of Liverpool Liverpool UK; ^4^ CSIR‐Forestry Research Institute of Ghana Kumasi Ghana; ^5^ Department of Natural Resources Management CSIR College of Science and Technology Kumasi Ghana; ^6^ Department of Zoology & Entomology University of Pretoria Pretoria South Africa; ^7^ School of Animal, Plant and Environmental Sciences University of the Witswatersrand Johannesburg South Africa

**Keywords:** adaptation, Blattodea, ectotherms, physiology, Termitoidae, thermal tolerance, tropics

## Abstract

1. The most diverse and abundant family of termites, the Termitidae, evolved in African tropical forests. They have since colonised grassy biomes such as savannas. These open environments have more extreme conditions than tropical forests, notably wider extremes of temperature and lower precipitation levels and greater temporal fluctuations (of both annual and diurnal variation). These conditions are challenging for soft‐bodied ectotherms, such as termites, to survive in, let alone become as ecologically dominant as termites have.
2. Here, we quantified termite thermal limits to test the hypothesis that these physiological limits are wider in savanna termite species to facilitate their existence in savanna environments.
3. We sampled termites directly from mound structures, across an environmental gradient in Ghana, ranging from wet tropical forest through to savanna. At each location, we quantified both the Critical Thermal Maxima (CT_max_) and the Critical Thermal Minima (CT_min_) of all the most abundant mound‐building Termitidae species in the study areas. We modelled the thermal limits in two separate mixed‐effects models against canopy cover at the mound, temperature and rainfall, as fixed effects, with sampling location as a random intercept.
4. For both CT_max_ and CT_min_, savanna species had significantly more extreme thermal limits than forest species. Between and within environments, areas with higher amounts of canopy cover were significantly associated with lower CT_max_ values of the termite colonies. CT_min_ was significantly positively correlated with rainfall. Temperature was retained in both models; however, it did not have a significant relationship in either. Sampling location explained a large proportion of the residual variation, suggesting there are other environmental factors that could influence termite thermal limits.
5. Our results suggest that savanna termite species have wider thermal limits than forest species. These physiological differences, in conjunction with other behavioural adaptations, are likely to have enabled termites to cope with the more extreme environmental conditions found in savanna environments and facilitated their expansion into open tropical environments.

## Graphical abstract

New information on the ecophysiology, and potential evolutionary strategies, of an incredibly important tropical taxa, termites; which are vital agents of decomposition in many tropical ecosystems.

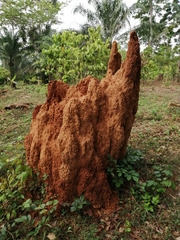

## 1 INTRODUCTION

In the terrestrial tropics, there are two dominant biomes: tropical forests and grassy biomes (savannas and grasslands; Mokany et al., 2006; Parr et al., 2014). The spatial distribution of these tropical environments has changed over time, as climatic conditions have varied. Notably, in the late Miocene and into the Pliocene, there was a major expansion of savanna grasslands (Cerling et al., 1993, 1997; Davies et al., 2020; Pagani et al., 1999), which coincided with the adaptive radiation of C_4_ grasses (Bouchenak‐Khelladi et al., 2014; Christin et al., 2013), and a recession of tropical forests. The expansion of savannas offered novel habitats for forest species to colonise, and while this provided opportunity for radiation of species, it also posed specific challenges. Understanding how species radiating from forest to savannas have overcome these challenges is vital for accurately predicting biogeographical history, making inferences of their current ecology, and predicting how these species may be affected by climate change.

Tropical forests and grassy biomes have markedly different environmental conditions. Forests have greater levels of canopy connectivity, rainfall, humidity (both relative humidity and vapour pressure deficit) and, typically, lower mean temperatures with a smaller thermal range (Nicholls & Wong, 1990; Sternberg, 2001; Tang et al., 2019). In contrast, tropical grassy biomes tend to have more extreme conditions with more variation than tropical forests, both spatially with more variable light and therefore temperature regimes, and also temporally over seasonal and diurnal time‐scales. Specifically, savanna conditions vary annually with pronounced dry seasons and large variation in temperature, humidity and rainfall across the year (D’Onofrio et al., 2019), but also diurnally, with the more open environment allowing for more extreme differences in environmental conditions throughout a 24‐hr period (due to higher levels of heat at ground level during the day, and a greater amount of heat loss during the night, when compared with forest; Hardwick et al., 2015).

Despite these environmental differences, a number of taxa have radiated from forests to savannas, adapting to the novel environmental challenges that savannas pose. For example, adaptations of grasses to open tropical environments (Bouchenak‐Khelladi et al., 2014; Christin et al., 2013; Pagani et al., 1999) include the notable dominance of C4 grasses in savannas, compared with the prevalence of C3 grasses in tropical forests (Adjorlolo et al., 2012; Solofondranohatra et al., 2018; Still et al., 2014). The generally more open canopy of savannas increases light availability for grass species which, coupled with the increased temperatures and decreased water availability, makes C_4_ photosynthesis more efficient than C_3_ (Solofondranohatra et al., 2018; Still et al., 2014). In contrast, in forests, the low understorey light levels and higher water availability favour C_3_ photosynthesis over C_4_ (Pearcy & Ehleringer, 1984; Solofondranohatra et al., 2018). Savanna grasses are also commonly taller and more tussocky, which allows them to compete for light more efficiently (Solofondranohatra et al., 2018). In addition, savanna species tend to be harder to decompose, with a higher a C:N ratio, which also facilitates burning (Simon & Pennington, 2012; Solofondranohatra et al., 2018). Forest grass species, on the other hand, tend to be creeping, low‐stature species, with large leaves to maximise light intake in the understorey, and have structures to minimise self‐shading (Solofondranohatra et al., 2018). Closely related plant species can therefore have very different evolutionary strategies to maximise their fitness in different tropical biomes.

Closely related animal species can also have radically different adaptations in different tropical environments. *Bicyclus* butterflies, important pollinators in both tropical African forest and savanna ecosystems (Dierks & Fischer, 2008), evolved a suite of traits when they colonised savannas from tropical forests, including diapause during the dry season, probably triggered by the lack of available suitable surfaces (grasses) for oviposition, which is in contrast to the forest species of *Bicyclus*, which do not have a period of diapause and are able to reproduce all year round (Halali et al., 2020).

However, little is known about how other invertebrate groups may have adapted to the novel environment that savannas present. The Termitidae, the most abundant and diverse group of termites (henceforth all mention of termites refers to the Termitidae family), also evolved inside tropical forests in central Africa (Eggleton, 2000; Aanen & Eggleton, 2005; Bourguignon et al., 2017). They have since radiated from forests into more open tropical environments (Aanen & Eggleton, 2005). Forests typically have higher generic and species richness than savannas, although some genera occur in both habitats. However, despite these shared genera, there are almost no shared species between the two environments (F. Evouna Ondo, unpubl. data), suggesting that while genera have radiated into savannas, they have since speciated and adapted to savanna environments. Similar to *Bicyclus* butterflies, savanna environments would have posed new challenges to colonising termite species. Termites being soft‐bodied ectotherms, making them extremely susceptible to desiccation (Heath et al., 1971; Willmer, 1982), would be challenged by the higher temperatures and lower humidity in savannas. Yet, despite the more extreme conditions, termites have colonised, diversified and become ecologically dominant in savannas, with a comparable level of biomass to large ungulates or elephants (Dangerfield et al., 1998; Støen et al., 2013). Termites are the dominant soil invertebrate across tropical ecosystems, contributing to multiple ecosystem processes including decomposition, nutrient cycling and bioturbation (Bottinelli et al., 2015; Fox‐Dobbs et al., 2010; Joseph et al., 2014; Jouquet et al., 2016; Turner, 2019). As ecosystem engineers, they influence these processes at landscape scales (Davies et al., 2014; Davies, Baldeck et al., 2016), altering the spatial distribution of nutrients and, as a result, plant species distributions (Davies, Baldeck et al., 2016; Davies, Levick et al., 2016; Joseph et al., 2014; Støen et al., 2013).

How termites have been able to colonise and to adapt to savanna conditions is currently uncertain. Termites have very different life‐history traits compared with other invertebrates (Poissonnier et al., 2018; Porter & Hawkins, 2001; Roisin, 2000); most termite species produce complex nest structures, and/or live underground (Jones & Oldroyd, 2006; Noirot & Darlington, 2000). These subterranean environments could protect and buffer termites from extreme environmental conditions, maintaining stable and ideal nest conditions (Gouttefarde et al., 2017; Jones & Oldroyd, 2006; Korb, 2003). The adaptation of nest structures to provide more efficient regulatory properties, such as altering the external shape to interact with the external temperature to maintain more stable internal conditions (Ocko et al., 2017; Singh et al., 2019; Vesala et al., 2019), may have facilitated the colonisation and success of forest termite species in savanna environments. However, while it is accepted that termite nests are vital in regulating some internal conditions, particularly gas flux (Knight, 2017; Ocko et al., 2017; Turner, 2001), whether nests also have thermoregulatory properties beyond the buffering nature of the soil, and are able to maintain steady internal temperatures, remains a topic of debate (Jones & Oldroyd, 2006; Korb, 2003; Turner, 1994).

Regardless of whether or not nest thermoregulation is present in termite species, termites forage outside the controlled environment of their nest, often on the surface, risking exposure to extreme environmental conditions (Traniello & Leuthold, 2000). Therefore, it follows that a key mechanism facilitating the expansion of termites into savanna is the evolution of wider physiological limits. Interspecific variation in thermal limits has been shown to occur across different latitudes in other invertebrate lineages (Diptera, *Drosophila*—Rajpurohit et al., 2008; Kellermann et al., 2012; various, but dominated by Coleoptera, Hymenoptera and Odonata—Sunday et al., 2014) and with exposure to higher temperatures (Diptera, *Drosophila‐*Parkash et al., 2008; Hymenoptera, Formicidae—Kaspari et al., 2015) with closely related species demonstrating significantly different thermal tolerances. The extension of termite physiological tolerances may have enabled them to withstand the more variable conditions and colonise savannas. However, few studies have documented the physiological limits of soil insects, including termites (but see, Woon et al., 2018), so the patterns observed in other studies may not hold across all insect orders.

In recent years, an increased focus on the thermal niche of species (Gvoždík, 2018; Kearney et al., 2010; Porter & Kearney, 2009; Sunday et al., 2014) has produced hypotheses to explain thermal performance, particularly given climate change concerns (Buckley et al., 2013; Clusella‐Trullas et al., 2011; Huey et al., 2012; Kearney & Porter, 2009; Sunday et al., 2011). These studies and hypotheses have contributed to developing the Thermal Adaptation Hypothesis (Angilletta et al., 2002; Angilletta, 2009; Huey & Kingsolver, 1989; Kaspari et al., 2015; Kingsolver & Gomulkiewicz, 2003), which states that species should be adapted to the thermal niche of their environment, therefore suggesting that species found in thermally more variable environments should have wider thermal ranges (Angilletta, 2009). In addition, the thermal adaptation hypothesis suggests that species in more thermally stable environments, as well as having narrower thermal ranges, should be operating closer to their thermal optimum (Angilletta, 2009). In the context of our study system, tropical forests have much more stable environmental conditions, whereas savannas have more extreme limits and more variation, so we would expect savanna species to have wider and more extreme physiological limits to adapt to their environment.

Here, we test the hypothesis that savanna termite species have wider physiological limits, which are likely to have facilitated their colonisation and speciation within savanna ecosystems. We quantify the thermal limits (critical thermal minima [CT_min_] and critical thermal maxima [CT_max_]) of the most abundant mound‐building termite species across tropical forest and savanna areas in Ghana, to test whether savanna species have more extreme physiological limits than forest species. We assess these data and discuss the applicability of the thermal adaptation hypothesis.

We predict that (a) termite species from savannas will have higher CT_max_ values than forest species, and that (b) they will have lower CT_min_ values. We correlated the physiological tolerances with climatic factors that change across the environmental gradient (from forest to savanna), temperature, rainfall and canopy cover, to determine which, if any, may be more strongly associated with physiological limits.

## 2 MATERIALS AND METHODS

### 2.1 Sampling locations

Termites were sampled from four locations in Ghana across two different biomes: tropical rainforest and Guinea savanna. The three forest locations were as follows: Bobiri Forest Reserve, a moist semi‐deciduous forest in the Ashanti Region, a matrix of forest patches that have been, or currently are, logged (all colonies sampled from fragments that have not been logged for at least 20 years); the Forestry Research Institute of Ghana (FoRIG) Campus, a moist semi‐deciduous forest patch located on the campus at Fumesua, near Kumasi, in the Ashanti Region; and the Assin‐Attandanso Resource Reserve side of the Kakum Conservation Area, a moist evergreen tropical forest that has not been logged since 1990 (Wiafe, 2016), in the Central Region. Termites were also sampled from the farmland surrounding Kakum National Park. Termites were sampled from two main sites in a single savanna park, Mole National Park, a large unfenced protected area in the Savannah Region. Sampling in all locations was conducted during the end of the dry season and the start of the wet season. Termites were sampled during the end of the dry season and start of the wet season at all sites: between January and March 2019 for the three forest sites, and June and July 2019 for the savanna site (Table 1).

**TABLE 1:** Summary statistics of the different sampling sites, with their locations and climatic conditions. The GPS coordinates are a representative location, at the approximate Centre of the sampling location. Climate data sourced from WorldClim2 (Fick & Hijmans, 2017)

Location	Vegetation type	GPS coordinates	Mean maximum temperature	Mean minimum temperature	Mean annual rainfall
Bobiri Forest Reserve	Rainforest	6.689 N, 1.342 W	31.3°C	18.5°C	1,306 mm
Forestry Research Institute of Ghana (FoRIG)	Rainforest	6.716 N, 1.529 W	31.4°C	19°C	1,357 mm
Kakum Farmland	Farmland (formerly forest)	5.568 N, 1.388 W	31°C	19.8°C	1,504 mm
Kakum National Park	Rainforest	5.573 N, 1.379 W	30.9°C	19.7°C	1,502 mm
Mole North	Savanna	9.334 N, 1.868 W	35.2°C	19.3°C	1,018 mm
Mole South	Savanna	9.286 N, 1.847 W	35.2°C	19.3°C	1,025 mm

### 2.2 Termite and environmental sampling

We sampled termites directly from termite mounds, identified to genus level (based on their diagnostic mound structures), which we located using directed searching, using the knowledge and expertise of field assistants. Two mounds were sampled per day, typically between 07:00 and 09:00 hr. Target mounds were broken open and we collected termites, as well as some mound material, and placed them into a cool box to protect the termites from external conditions and reduce stress that could affect their thermal tolerances while being transported to the laboratory. The sample from each colony was placed in a separate container to prevent interaction between the two colonies. Where possible, both worker and soldier castes were sampled. The time between sampling and experimentation was reduced as much as possible (mean = 122.6 min, *SD* = ±81.4 min). The large standard deviation is attributed to having a single collection, but running two experiments, per day. The same colonies that were sampled were used in two experiments, one testing thermal maximum and one testing thermal minimum (the order of which was decided randomly); hence, the individuals used in the second experiment were kept inside the cool box (which was out of direct sunlight) while the first experiment took place.

We photographed each mound to assist with species identification. Canopy cover above each sampled mound was quantified using a hemispherical fish‐eye lens (Canon EOS 70D camera, Sigma 4.5 mm f/1:2.8 Circular Fisheye Lens). For large mounds, the image was taken directly above the apex of the mound, but with small mounds (<75 cm in height) the image was taken above the mound at a height of 75 cm due to the height of the stabilising monopod. Due to variable light conditions when the images were taken, we did not standardise ISO, shutter‐speed or aperture, although variation in these settings was reduced as much as possible. Variable light conditions and camera settings were corrected using Gap Light Analyser software (GLA; Frazer et al., 1999). The approximate leaf‐area index above each mound was then calculated as a measure of canopy cover. Leaf‐area index is a measurement of the quantity of leaf area in a canopy per unit area of ground, and ranges from 0 (no leaf cover) to 7 (complete leaf cover).

### 2.3 Thermal tolerance experiments

We used similar methods to those outlined in Bishop et al. (2018) to estimate the thermal tolerance of the termites. In all, 14 individuals from each of two colonies (28 in total) were tested per experimental run. Only individuals of the helper castes, workers and soldiers, were used in the experiments. Each termite was placed into a separate microcentrifuge tube. The tubes were then placed into a dry heat bath (Tropicooler 260,014–2, Boekel Scientific, Feasterville, PA, USA; temperature range of −19°C–69°C and an accuracy of ±1°C). The heat bath contained two aluminium inserts with each consisting of 14 wells, each of which held a single microcentrifuge tube. Individuals from two colonies and different castes were randomly allocated to different wells to remove potential equipment bias. The two colonies sampled each day were used in each experimental run (CT_max_ and CT_min_) and each colony was tested for both CT_max_ and CT_min_; however, each individual termite was only used in one experiment.

The temperature within each microcentrifuge tube was acclimated for 15 min. CT_max_ experiments had an acclimation temperature of 30°C, and CT_min_ had an acclimation temperature of 24°C. After the acclimation period, the temperature was changed by 1°C (raised for a CT_max_ experiment, and lowered for CT_min_), and maintained at each new temperature for a further 3 min. Following this 3‐min period, we checked each termite for the onset of a heat or chill coma. Heat coma onset was considered to have occurred when the individual lost muscle coordination; if this occurred, it was recorded as the CT_max_ of that termite. A chill coma was considered to have occurred when there was a complete lack of movement despite flicking the microcentrifuge tube; if this occurred, it was recorded as the CT_min_ of that termite. When we noticed the termite had lost muscle coordination (CT_max_) or had stopped moving (CT_min_), the temperature was recorded as the thermal limit of that individual termite. The experimental run was ended when the thermal limit of all 28 termites were recorded.

### 2.4 Termite identification

**FIGURE 1:**
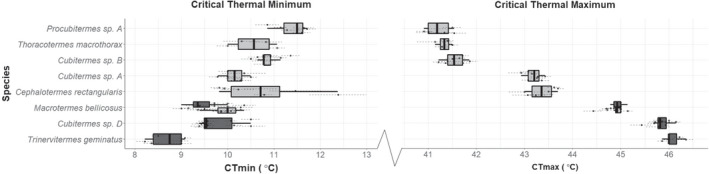
Relationship between termite species thermal limits and the habitat they were sampled from. Species are ordered from lowest CT_max_ (top) to highest CT_max_ (bottom). Colour of the boxplot denotes the location the termites were sampled, dark grey from savanna and light grey from forest. Macrotermes bellicosus was sampled from both savanna and forest environments, and so it has distinct boxplots for each community. The points represent the estimated thermal limit of a unique termite colony, and the dotted lines represent the standard error around the colony average. Central thick line of the boxplot represents the species median thermal limit, the box represents the interquartile range, and the whiskers the upper and lower adjacent values

Termite colonies were identified to genus level in the field based on their mound structure. All termites used in experimental runs were preserved in 70% ethanol and brought to the UK. We took an individual from each colony and DNA was extracted from each sample. The *COII* mitochondrial gene was amplified and sequenced using Sanger Sequencing at the Natural History Museum, London. Each sequence was matched with the most closely related sequence on NCBI using BLAST (Johnson et al., 2008). If the sequences had a unique high likelihood identity match (>98%) with a single species described on the NCBI database, that sample, and therefore the colony it was sampled from, was considered identified as that species. If there was not a unique high likelihood identity match, the sequences (and therefore the colonies from which each sample was taken) were grouped and labelled as an unspecified species within a genus (e.g. *Cubitermes sp*. *A*, Figure 1). After identification, species that did not have at least three replicates per sampling location were removed from the dataset (19 colonies removed, present in online dataset). Since the genetic identification was conducted, the genus *Cubitermes* was split into several genera (Hellemans et al., 2021). However, there were limited sequence data for the new classifications so we have treated all the new genera as *Cubitermes* sensu *lato*.

### 2.5 Climate data

Climate data were sourced from the WorldClim2 dataset (Fick & Hijmans, 2017) using the raster package (Hijmans et al., 2020) in the R statistical software version 3.6.2 (R Core Team, 2019). WorldClim2 gets average measurements of climatic variables each month, based on an interpolated dataset between 1970 and 2000. We extracted measurements of average monthly rainfall, average monthly maximum temperature and average monthly minimum temperature to the highest resolution possible (30 arc seconds, equivalent to approximately 1 km^2^) for each mound we sampled. From these data, we obtained average daily rainfall, highest average maximum temperature and lowest average minimum temperature for the area around each colony centre.

### 2.6 Statistical analysis

After genetic identification, eight species had a sample size of three colonies and were included in the statistical analyses. Three of those species were sampled from the two sites within the savanna location (Mole North, Mole South, Table 1), and six species were sampled from the forest locations (FoRIG, Bobiri, Kakum Forest and Kakum Farmland, Table 1). One species, *Macrotermes bellicosus*, was sampled in both savanna and forest locations, but it was only found in the forest environment that was heavily disturbed, in Kakum Farmland (Table 1).

All statistical analyses used the R statistical package version 3.6.2 (R Core Team, 2019). We calculated the estimated colony CT_max_ and CT_min_ by taking the average of the thermal tolerance of each termite within that colony that was used in an experimental run. Initially, basic Welch’s two‐sample t‐tests were run to compare the thermal tolerances of species assemblages sampled in forest and savanna environments (termites sampled from Kakum Farmland were not included, Table 1). A t‐test was also used to compare *Macrotermes bellicosus* thermal tolerances in two different environment types (farmland and savanna), and a one‐way analysis of variance test was used to compare the thermal tolerances of the three *Cubitermes*‐group species sampled. Tukey’s HSD post‐hoc statistics were used for pairwise comparisons of the species thermal tolerances.

To test whether environmental variables affect termite colony thermal limits, mixed‐effects models were fitted using the R package lme4 (Bates et al., 2019), with a separate model for CT_max_ and CT_min_. Maximal models tested the interactive effects of canopy cover, average daily rainfall (which was rescaled from annual rainfall) at the mound and temperature at the mound (maximum temperature for the CT_max_ model and minimum temperature for the CT_min_ model). A single random effect of colony location (as listed in Table 1, Figure S1) was used to account for additional unmeasured environmental differences. Species was not included as a random effect (crossed or nested) because there was little overlap in species composition between sampling locations, and so both site and species would therefore explain the same variation, and there was not enough data to prevent non‐convergence.

To understand which of the fixed effects and their interactions best explained the variation in termite thermal tolerances, two lists of candidate models were generated (one for CT_max_ and one for CT_min_) using the ‘dredge’ function from the mumin package (Bartoń, 2019). This takes the maximal model and systematically removes variables and/or interactions until all possible combinations have been tested, and compared these complete lists of models using the package aiccmodavg (Mazerolle & Linden, 2019). The model with the lowest corrected AIC (AICc) value was obtained, and the other models with a difference in AICc (ΔAICc) of less than two were retained to inform the final ‘full’ model (Tables S1 and S2; Burnham & Anderson, 2002). Model averaging, using the candidate lists of the model with the lowest AICc value and all those with ΔAICc <2, was used to identify the best predictor variables across the models. The relative importance of these predictors was calculated as the sum of their relative Akaike weights from all models in which they appear; thus, models with lower AICc values contribute more to the final ‘full’ model. A series of *F* tests was also conducted to verify the significance of the retained parameters on the thermal limits of the termite species tested in the final models. This process was conducted for two separate model sets, one testing CT_min_ and the other CT_max_, generating two ‘full’ models.

## 3 RESULTS

A total of 2042 termite specimens from 71 colonies and eight species were used in the final analyses (1022 termites for CT_max_ experiments and 1020 termites for CT_min_ experiments). There was a large amount of interspecific variation in thermal tolerance, with species averages varying by 4.86°C for their CT_max_ and 2.96°C for their CT_min_ (Figure 1). Savanna species had significantly higher CT_max_ values on average than forest species (*t* = −17.309, *p* < 0.001,   *df* = 52.37, Figure 1a), and significantly lower CT_min_ values on average than forest species (*t* = 8.7035, *p* < 0.001,   *df* = 53.84, Figure 1b); as a result, savanna species had wider thermal limits than forest species. The three *Cubitermes*‐group species tested had significantly different CT_max_ values (*p* < 0.001, *F*_2,26_ = 1424, Figure 1a, S3) and CT_min_ values (*p* < 0.001, *F*_2,26_ = 29.24, Figures 1b, S2), with all species having significantly different thermal niches from one another (Tukey’s HSD; Figures S2 and S3). *Macrotermes bellicosus* from multiple environments was tested (farmland in the forest zone and savanna), but CT_max_ did not differ among the two habitats (*p* = 0.805, *t* = 0.255, *df* = 7.775, Figure 1a). *M. bellicosus* did, however, have significantly different CT_min_ values (*p* = 0.008, *t* = 3.132, *df* = 12.266, Figure 1b), with savanna colonies having lower CT_min_.

### 3.1 Critical thermal maximum

**FIGURE 2:**
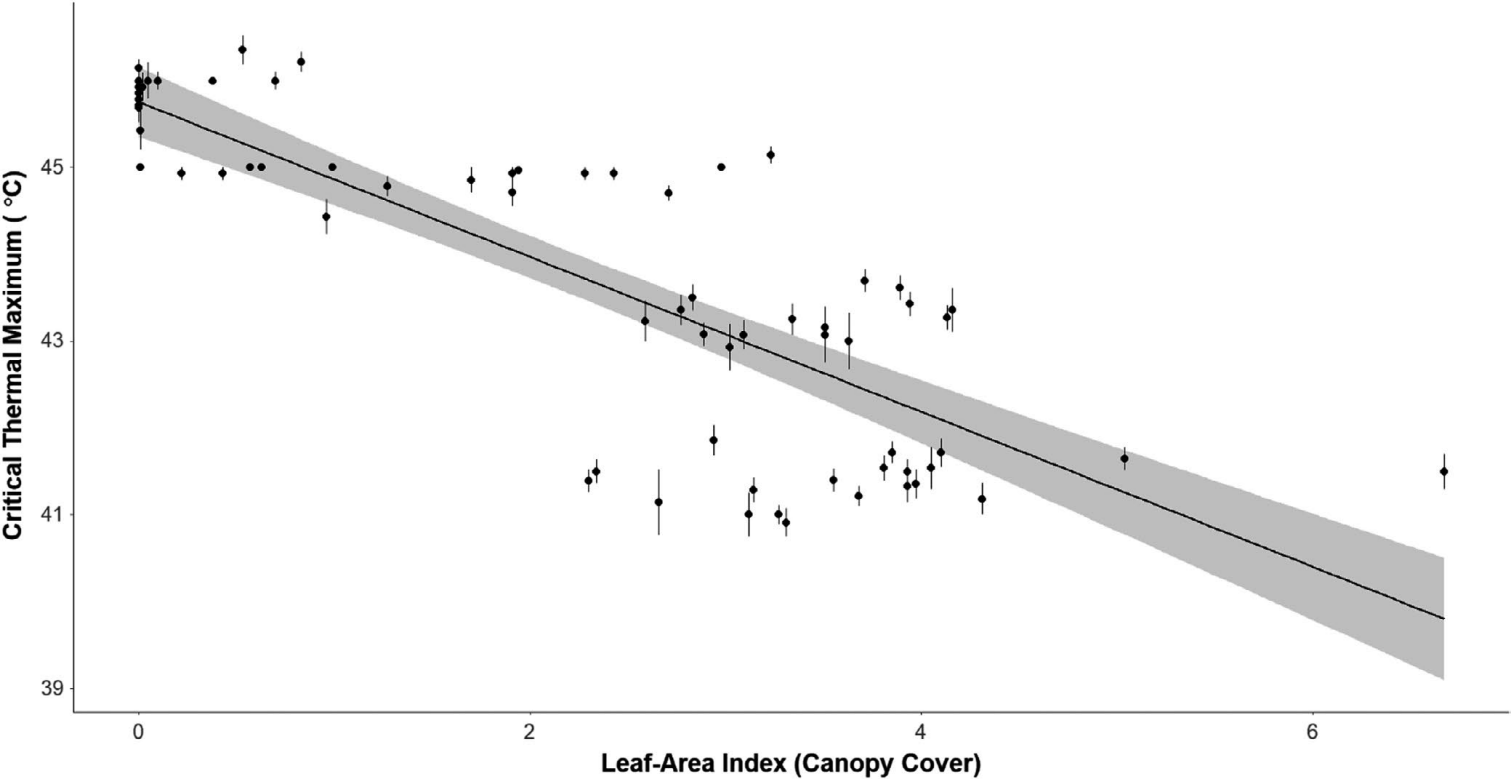
Significant negative correlation between the estimated critical thermal maximum of termite colonies and the canopy cover above the colony centre (*p* = 0.001), as predicted by a linear mixed‐effects model (with sampling location as the random effect). Leaf‐area index is the measurement of canopy cover used, with higher leaf‐area index representing higher canopy cover above the mound. Each point represents the estimated thermal maximum of a unique termite colony across all locations sampled, the thermal maximum is averaged from each individual termite that was used in the experiment and the line surrounding each point represents the standard error around this mean. The thick black line represents predicted relationship based on the model, with the shaded grey area representing the 95% confidence interval around these predictions

The full model retained all three fixed effects (canopy cover, maximum temperature and average daily rainfall; Table 2), but the interaction between the terms was removed during the model selection process. Of the three remaining variables, only canopy cover had a significant negative correlation with colony CT_max_ (*z* = 3.138, *p* = 0.001, *df* = 63.470; Figure 1a; Table 2), suggesting that as the canopy cover above termite mounds increases, the average critical thermal maximum of the termite colony decreases (Figure 2). Despite being retained in the full model, neither maximum temperature (*z* = 1.215, *p* = 0.358, *df* = 3.900; Table 2) nor rainfall (*z* = 0.068, *p* = 0.964, *df* = 3.689; Table 2) showed a significant correlation with colony CT_max_. The single random effect of colony location (Table 1) explained 48.6% of the variation in the model.

**TABLE 2:** ‘Full’ averaged model coefficients and their significance as produced by the aiccmodavg package (Mazerolle & Linden, 2019) modelling termite CT_max_ change as a function of environmental factors. The full model fitted the following fixed effects; average maximum temperature at the mound, average daily rainfall at the mound and leaf‐area index at the mound (a measure of canopy cover), and a single random effect of sampling location to predict the critical thermal maximum of termite colonies. Asterisks indicate level of significance

Model parameter	Full model‐averaged coefficient	Lower CI	Upper CI	*z*‐value	*p*‐value
*Intercept*	31.984	13.772	50.196	1.756	0.079
Leaf‐area index (Canopy Cover)	−0.367	−0.483	−0.250	3.138	0.001
Maximum temperature	0.385	−0.034	0.804	0.919	0.358
Average daily rainfall	0.063	−1.340	1.466	0.045	0.964

### 3.2 Critical thermal minimum

**FIGURE 3:**
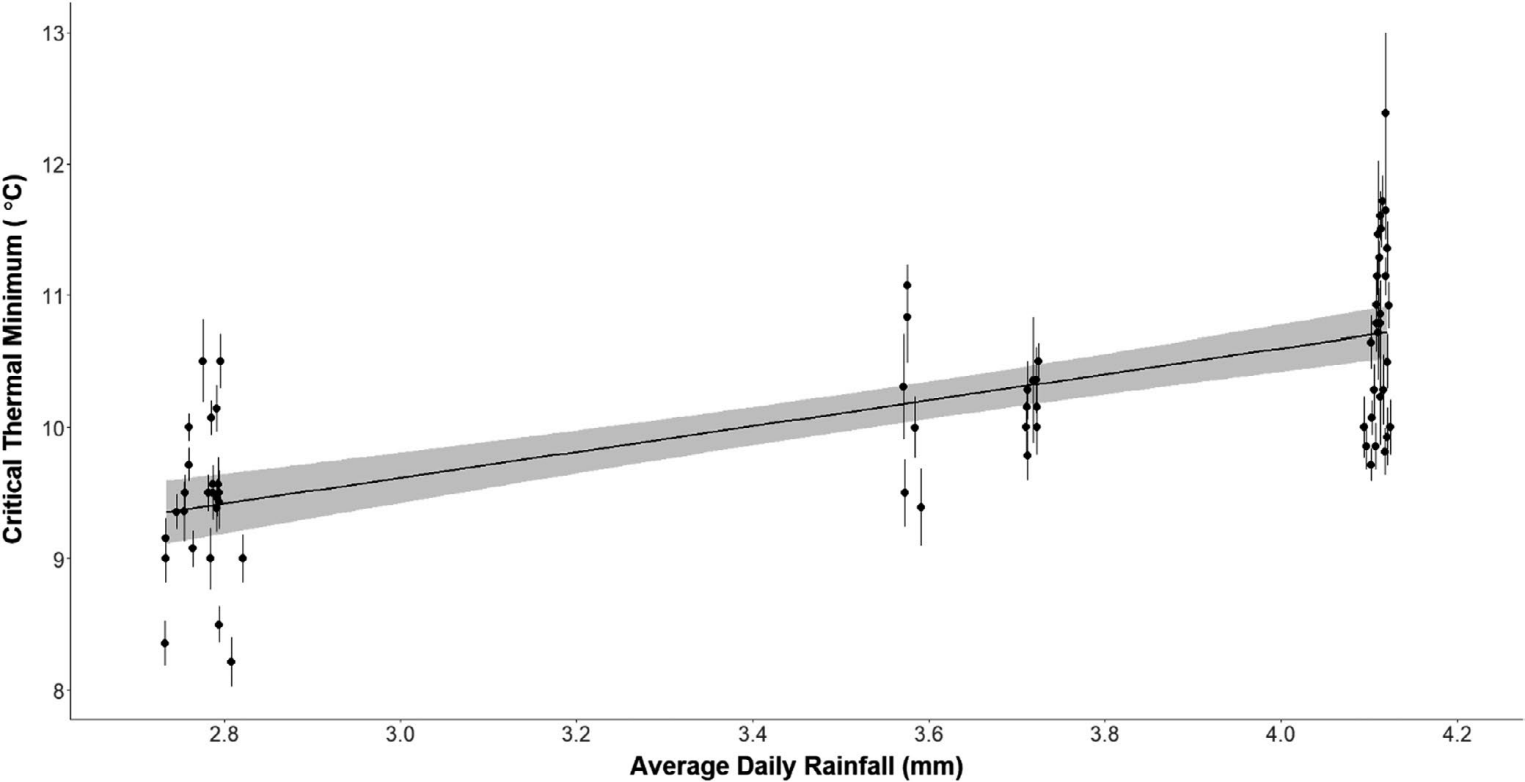
Significant positive correlation between average daily rainfall at the mound (data obtained from WorldClim2, Fick & Hijmans, 2017), and the estimated critical thermal minimum of termite colonies (*p* = 0.007), as predicted by a linear mixed‐effects model (with sampling location as the random effect). Each point represents the estimated thermal minimum of a unique termite colony across all locations sampled, the thermal minimum is averaged from each individual termite that was used in the experiment, and the line surrounding each point represents the standard error around this mean. The thick black line represents predicted relationship based on the model, with the shaded grey area representing the around these predictions

The full critical thermal minimum model retained two fixed effects (canopy cover and average daily rainfall; Table 3), and it also removed all interactions between the variables. Unlike in the CT_max_ model, canopy cover was not significantly correlated with CT_min_ of termite colonies (*z* = 0.269, *p* = 0.788, *df* = 59.671; Table 3); however, average daily rainfall was significantly positively correlated with CT_min_ (*z* = 2.679, *p* = 0.007, *df* = 4.504; Figure 3; Table 3). Colony location (Table 1) explained 39.6% of variation in the model.

**TABLE 3:** ‘Full’ averaged model coefficients and their significance as produced by the aiccmodavg package (Mazerolle & Linden, 2019) modelling termite CT_min_ change as a function of environmental factors. The full model fitted the following fixed effects; average minimum temperature at the mound, average daily rainfall at the mound and leaf‐area index at the mound (a measure of canopy cover), and a single random effect of sampling location to predict the critical thermal minimum of termite colonies. Minimum temperature was not retained in the final model. Asterisks indicate level of significance

Model parameter	Full model‐averaged coefficient	Lower CI	Upper CI	*z*‐value	*p*‐value
*Intercept*	6.878	5.714	8.042	5.910	2e^−16^
Average daily rainfall	0.885	0.555	1.216	2.679	0.007
Leaf‐area index (Canopy Cover)	0.011	−0.030	0.052	0.269	0.788

## 4 DISCUSSION

Our findings support the hypothesis that savanna termite species have more extreme physiological tolerances, and these tolerances are likely to have been a key mechanism that has allowed termites to colonise savanna environments. As predicted, savanna species had a significantly higher CT_max_ than forest species. In addition, CT_max_ had a significant negative correlation with canopy cover (Figure 2). As the canopy cover decreases, the amount of sunlight reaching the soil surface and the surface of the mound increases, which will increase the temperature of the air, soil surface, and potentially within the mound and foraging areas of termites (Hardwick et al., 2015). Canopy cover in our savanna environments (average 26.6 ± 5.8%) was much lower than that found in tropical forests (84.7 ± 3.1%). To cope with higher day temperatures in savannas, the termites present will require mechanisms such as increased thermal tolerance. Our data support this idea, not only due to the positive correlation between canopy openness and CT_max_, but also among closely related species (e.g. of the *Cubitermes‐*group) in which species in the more open and extreme savanna habitat had more extreme thermal limits (Figure 1a and Figure 1b). *Cubitermes‐*group species are soil feeding termites, so do not need to leave the protection of the soil to forage for food and so have a reduced interaction with ambient conditions (when compared to other species), yet they have significantly different thermal tolerances between species from the two environments. If these species had developed extensive mound thermoregulatory mechanisms such that the environmental conditions experienced by the termites were similar, we would expect closely related species to have similar thermal tolerances, irrespective of the location they were sampled from; this is not the case with the *Cubitermes‐*group species we sampled, as closely related species have significantly different thermal tolerances.

Surprisingly, despite savanna species having a significantly lower CT_min_ than forest species, CT_min_ was not significantly correlated with canopy cover. We expected that lower canopy cover in savannas would allow the ground surface to cool more rapidly at night, and result in lower temperatures. Consequently, we expected canopy cover to positively correlate with CT_min_, as termites in locations with lower canopy cover will experience lower temperatures at night. This is not what was found; CT_min_ was significantly positively correlated with average daily rainfall (Figure 3). We are unsure about the underlying process linking lower CT_min_ with reduced rainfall. The species from the savanna regions had lower CT_min_ values, which has lower rainfall and therefore lower cloud cover, which would increase heat loss and result in lower temperatures; however, minimum temperature was included in the model and had no significant effect. More sampling is required, additional species from more locations along the environmental gradient, including between the forest and savanna environments, and further north of our savanna sites (in locations which would have higher day temperatures and lower night temperatures) to properly tease apart these relationships.

Canopy cover was expected to have a significant relationship with CT_min_ because some termite species forage during cool parts of the day, including night time, which would put pressure on the CT_min_ of foraging individuals, and that pressure would be greater in areas with low canopy cover where the less heat would be retained overnight. For example, the specialist savanna grass‐feeder, *Trinervitermes*, typically forages at dusk, dawn or during the night (Adam et al., 2008). Our data support this: *Trinervitermes germinatus* had the lowest CT_min_, which would be expected for coping with the lower temperatures experienced during foraging at night. Other studies have shown that some savanna species (e.g. *Macrotermes* sp.; Darlington, 1982) forage during cooler parts of the day or when overcast (Evans & Gleeson, 2001; Parr pers obs); however, these studies are few in number and do not cover the species studied here. In general, behavioural avoidance of one thermal extreme (e.g. in the case of *Trinervitermes*, hot conditions) may reduce selection pressures on the physiological tolerance of that extreme (e.g. CT_max_), but not necessarily the opposite extreme. This interplay between behavioural and physiological adaptations represents an important avenue for future study.

Mean site temperature did not have a significant effect on thermal tolerance at maximum or minimum temperatures. This may be because the temperature data used in both models were interpolated temperature of air (Fick & Hijmans, 2017), which is unlikely to be the best measure of the conditions that termites would experience. Due to their small size, terrestrially foraging termites forage in the thermal boundary layer, immediately above a surface of, in this case, the ground. The thermal boundary layer has very different conditions from the recorded air temperature, both during the day (where it is likely to be super‐heated) and at night (retaining more heat due to proximity to soil). Boundary layer temperature, or ground surface temperature, would be a more accurate representation of the true thermal conditions that termites experience, but unfortunately these data were not available at a fine‐enough spatial scale. So, while it is likely that air temperature and ground temperature are correlated, and lack of ground temperature does not invalidate these results, the models would be improved by access to these data.

There was more intraspecific variation in CT_min_ than CT_max_, which was possibly associated with measurement error. The protocol for recording CT_min_ used for other insects, such as ants (Bishop et al., 2018; Kaspari et al., 2015), involves recording the temperature at which all movement ceases. However, termites are much less mobile than ants, making it extremely difficult to tell the difference between the onset of a chill coma (Hazell & Bale, 2011) and the true CT_min_. Therefore, whereas the values described here are biologically relevant, as termite fitness would be severely negatively affected if temperatures were to drop to near these recorded ‘CT_min_’ values, the values recorded likely contain measurement error. An additional method to test for signs of life in other insects is to shine a laser pen into the eye of the individual in an attempt to elicit a head‐movement response. This method is not possible with termites, as the helper castes of almost all species lack eyes, so the only stimulus that could be used was flicking the test tube. Another source of error is that a ‘no movement’ reaction in a living individual is impossible to distinguish visually from a dead individual. Consequently, these measurements may slightly underestimate true CT_min,_ resulting in greater intraspecific variation than the true range. However, the measurement errors are likely to be random, rather than systematically correlated with environmental variables. Moreover, despite the measurement inaccuracies, CT_min_ correlated significantly with rainfall, and the differences in CT_min_ between the species were pronounced.

Differences in humidity, however, could be a source of variation in these results. While conditions between sampling and experimentation were controlled as much as possible, maintaining conditions close to internal nest conditions was impossible. Termites, being soft‐bodied ectotherms, are extremely susceptible to changes their environment, particularly changes in humidity. As we have shown in the past (Woon et al., 2018), the reduction of humidity could significantly affect their fitness. Therefore, such changes in the humidity in the test tubes during experimentation could affect the thermal tolerances of the individuals tested. We had no method of recording the humidity of the test tube, but the difference between the internal nest conditions of termite species and the test conditions would be likely to be larger in savannas. The size of the effect of test tube humidity on termite critical thermal maxima, however, is unknown, so understanding how changes to air conditions (other than temperature) affect termite fitness is an important avenue of future study. The effect of the reduction in humidity, and how this interacts with changes in temperature, is relevant for understanding how a broad spectrum of changes under global warming may affect termite fitness.

Although we think it extremely unlikely that there are mound thermoregulatory mechanisms in the *Cubitermes*‐group species we sampled because of their very different thermal limits, our data are equivocal as to whether there is mound thermoregulation in *Macrotermes bellicosus* mounds. This is a topic of much debate (Korb, 2003; Jones & Oldroyd, 2006; Gouttefarde et al., 2017; Turner, 1994; Turner, 2001), because although large termite mounds can alter the internal temperature when compared with external air conditions (Korb & Linsenmair, 2000; Ndlovu et al., 2021; Ndlovu & Pérez‐Rodríguez, 2018; Vesala et al., 2019), it has been argued that the energy required to alter the internal nest conditions from the surrounding soil temperature would be extremely high (Turner, 1994; Turner, 2001). So although *M. bellicosus* had the same CT_max_ in all locations sampled despite environmental conditions varying among habitats, this does not provide sufficient evidence for thermoregulatory mechanisms within their mounds. In addition, we sampled *M. bellicosus* from their native region (savanna) and further south of their ancestral distribution, areas which have less extreme environmental conditions, particularly a much lower maximum temperature, which would not put any additional pressure on their thermal limits. Improved knowledge of whether thermoregulation is present in *Macrotermes* mounds could come from comparisons of thermal tolerances of *M. bellicosus* (and indeed other *Macrotermes* species) from multiple savanna environments, with varying climatic conditions (e.g. those with hotter and drier climates).

As predicted by the thermal adaptation hypothesis, the forest species had much narrower thermal limits than the savanna species (Angilletta, 2009). Forests have more stable temperatures (less diurnal and annual variation), so the theory posits that the termite species found there should have narrower thermal limits than the savanna species. This prediction of the thermal adaptation hypothesis holds true, and it is likely that the stability, and lower extremes, of forest environments have contributed to narrower thermal limits (and therefore thermal niches) of forest termite species. How close the current temperatures are to termite thermal limits is not known, however, due to the imprecise nature of the temperature data. Obtaining more accurate temperate data, which measures surface temperature or boundary layer temperature, over a finer spatial scale, and recorded from a more recent time period would allow us to make a more accurate prediction of the resilience of termite assemblages to a warming climate. Understanding how close the thermal conditions that termites experience are to their thermal limits, and to their thermal optima, is vital in making this prediction.

A number of other studies have demonstrated environments with more variable climatic conditions tend to contain species with wider thermal limits (e.g. ants—Bujan et al., 2016; dung beetles—Gaston & Chown, 1999; tadpoles—Gutiérrez‐Pesquera et al., 2016), similar to our findings. However, our results also suggest that the thermal limits of termite species are strongly correlated, that is, a species with a high CT_max_ is also likely to have a low CT_min_. This suggests that the widening of savanna termite thermal ranges occurs at both ends of their thermal range, with both limits being selected by their environment. Whether the widening of these limits are separate, or intrinsically linked, would be an interesting direction for future study.

Termites are extremely efficient ecosystem engineers, managing the internal conditions of their nests and foraging areas to be more suitable for their own exploitation (Dangerfield et al., 1998; Jouquet et al., 2011). While they alter a number of conditions, such as light penetration and nutrient content of the soil (e.g. through the construction of physical structures such as mounds and sheeting; (Davies, Levick, et al., 2016; Ndiaye et al., 2004), the most pronounced alteration they make is through the hydrology of their local environment (Mando et al., 1996; Turner, 2019; Turner et al., 2006). Termites, and particularly those that operate over large spatial scales such as *Macrotermes* species, are able to access water from deep in the soil profile (Holt & Lepage, 2000; Jouquet et al., 2011; Turner et al., 2006) and maintain extremely high levels of moisture in the walls of their nests and foraging locations. Termites and their structures have been shown to alter the spatial distribution of water at an environment‐wide scale, altering seedling survival rate (Ashton et al., 2019) and changing the plant species that grow on and around termite mounds (Davies, Levick, et al., 2016; Jouquet et al., 2016). As a result, it is argued that termite groups such as *Macrotermes* can live in more arid environments than they otherwise should be able to. This might, in turn, change the pressures on their physiological limits, including their thermal tolerance.

This ability to alter their environment might also feedback, causing an evolutionary response as outlined by niche construction theory (Laland et al., 2016; Odling‐Smee et al., 2013). Namely, the ability of termites to modify the conditions they experience may change the pressures presented on their physiological limits, thus causing an evolutionary response (Matthews et al., 2014). The modification of their local environment is likely to reduce the environmental pressure, but it is unknown as to how, or to what extent, this may affect their physiological limits. Understanding how environmental water availability and termites' ability to control the hydrology and humidity of their altered environments interact with their physiological tolerances is required, particularly in the face of climate change.

Our results suggest that for termites, the most important soil macroinvertebrates in savanna environments (Davies, Levick, et al., 2016; Jouquet et al., 2016), a key mechanism that has facilitated their colonisation of the seasonal, climatically extreme environments of savannas from forests is the widening of their thermal tolerances. However, temperature is not the only variable that changes along an environmental gradient from forest to savanna. Food type, food availability, soil mineral content (and pH), soil moisture content, predator diversity, predator abundance and, as mentioned previously, humidity, are a few of the factors that could change along the gradient, and therefore influence termite species distributions. Some of these would contribute to the large proportion of variation that is explained by the random effect of sampling location in both models, making them promising avenues for future research.

Understanding how termite physiology and behaviour changes across environmental gradients such as this one is particularly important in the context of climate change. The thermal adaptation hypothesis predicts that species in more stable environments will be more heavily impacted by warming conditions, due to those species operating closer to their thermal maximum (Angilletta, 2009), and be more susceptible to extinction. This suggests the forest species here may be more vulnerable to rising temperatures associated with climate change, but more research is needed to understand the complexities of physiological change, how it affects termite distributions and how they might be altered by the changing climate.

Our data suggest that the widening of termite thermal tolerances has occurred in species that have established in areas of more extreme climatic conditions, and that is likely a large factor in allowing termites to persist, speciate and become ecologically dominant in arid, tropical environments. Whether the widening of these physiological limits will make them more resilient to climate change is a question that remains to be answered.

## CONFLICT OF INTEREST

All authors declare no conflict of interest.

## AUTHORS' CONTRIBUTIONS

J.S.W., C.L.P. and P.E. conceived the ideas and designed the methodology; J.S.W., C.L.P. and S.A.‐B. collected the data and facilitated the data collection; J.S.W. designed the analysis and carried out all the analyses. All authors contributed critically to the drafts and gave final approval for publication.

## Supporting information


Supinfo

## Data Availability

The data used in this study are archived on Dryad Digital Repository; 10.5061/dryad.v41ns1rxz (Woon et al., 2022).
