# Supplementary material for: Termites have wider thermal limits to cope with environmental conditions in savannas
Source: J Anim Ecol. 2022 Feb 24;91(4):766–79. doi: 10.1111/1365-2656.13673 (PMC9307009; doi:10.1111/1365-2656.13673)
Supplement: Supplementary file 1 — Supinfo [file JANE-91-766-s001.docx]

| **Model Number** | **Leaf-Area Index (1)** | **Maximum Temperature (2)** | **Rainfall (3)** | **1 : 2** | **1 : 3** | **2 : 3** | **1 : 2 : 3** | **k** | **log-Likelihood** | **R^2^** | **AICc** | **∆AICc** |
| --- | --- | --- | --- | --- | --- | --- | --- | --- | --- | --- | --- | --- |
| **1** | + | + |  |  |  |  |  | 5 | -93.495 | 0.761 | 193.186 | 0.000 |
| **2** | + |  | + |  |  |  |  | 5 | -92.779 | 0.755 | 194.946 | 1.760 |
| **3** | + | + | + |  |  |  |  | 6 | -91.540 | 0.762 | 195.154 | 1.968 |
| **4** | + |  |  |  |  |  |  | 4 | -94.535 | 0.745 | 195.442 | 2.256 |
| **5** | + | + |  | + |  |  |  | 6 | -95.469 | 0.761 | 195.475 | 2.289 |
| **6** | + |  | + |  | + |  |  | 6 | -93.544 | 0.755 | 197.357 | 4.171 |
| **7** | + | + | + | + |  |  |  | 7 | -93.512 | 0.763 | 197.499 | 4.313 |
| **8** | + | + | + |  | + |  |  | 7 | -92.329 | 0.762 | 197.681 | 4.495 |
| **9** | + | + | + |  |  | + |  | 7 | -90.519 | 0.761 | 197.892 | 4.707 |
| **10** | + | + | + | + | + |  |  | 8 | -91.543 | 0.765 | 199.306 | 6.120 |
| **11** | + | + | + | + |  | + |  | 8 | -92.464 | 0.761 | 200.424 | 7.238 |
| **12** | + | + | + |  | + | + |  | 8 | -91.272 | 0.761 | 200.628 | 7.442 |
| **13** |  | + |  |  |  |  |  | 4 | -96.757 | 0.723 | 201.331 | 8.145 |
| **14** | + | + | + | + | + | + |  | 9 | -90.443 | 0.765 | 202.007 | 8.821 |
| ***Intercept Only*** |  |  |  |  |  |  |  | 3 | -97.847 | 0.707 | 203.038 | 9.852 |
| **15** |  |  | + |  |  |  |  | 4 | -96.045 | 0.716 | 203.076 | 9.890 |
| **16** |  | + | + |  |  |  |  | 5 | -94.469 | 0.725 | 203.115 | 9.929 |
| **17** | + | + | + | + | + | + | + | 10 | -89.953 | 0.765 | 204.792 | 11.606 |
| **18** |  | + | + |  |  | + |  | 6 | -93.309 | 0.726 | 205.258 | 12.072 |

Table S1 – AIC output from the ‘dredge’ function comparing the relative strength of potential models explaining the relationship between termite CTmax and climatic changes in the environment in which the termites are found.

Notes: The model parameters are in columns two through eight, and the parameters included in each of the models (numbered in the first column) are denoted by a “+” sign, and a light grey highlight. Each individual parameter is numbered, and the interactions between these parameters are indicated by these numbers in columns five to eight. The number of degrees of freedom lost in the model (k), the log-likelihood of the model, the R^2^ of the model, the AICc value, and the difference in the AICc value of the model and the model with the lowest AICc (∆AICc) are also shown. In all models, the random effect of sampling location is included. Please note, rainfall was rescalled from annual rainfall to average daily rainfall to account for differences in size with the other explanatory variables.

| **Model** | **Leaf-Area Index (1)** | **Minimum Temperature (2)** | **Average Daily Rainfall (3)** | **1 : 2** | **1 : 3** | **2 : 3** | **1 : 2 : 3** | **k** | **Log-Likelihood** | **R^2^** | **AICc** | **∆AICc** |
| --- | --- | --- | --- | --- | --- | --- | --- | --- | --- | --- | --- | --- |
| **1** |  |  | + |  |  |  |  | 4 | -56.434 | 0.595 | 119.456 | 0.000 |
| **2** | + |  | + |  |  |  |  | 5 | -58.061 | 0.597 | 121.281 | 1.825 |
| **3** |  | + | + |  |  |  |  | 5 | -56.341 | 0.592 | 122.188 | 2.732 |
| **4** | + |  | + |  | + |  |  | 6 | -59.256 | 0.600 | 123.165 | 3.709 |
| ***Intercept Only*** |  |  |  |  |  |  |  | 3 | -58.792 | 0.559 | 123.222 | 3.766 |
| **5** | + | + | + |  |  |  |  | 6 | -57.997 | 0.595 | 124.168 | 4.712 |
| **6** |  | + | + |  |  | + |  | 6 | -54.787 | 0.594 | 124.260 | 4.804 |
| **7** | + |  |  |  |  |  |  | 4 | -60.362 | 0.562 | 124.973 | 5.517 |
| **8** |  | + |  |  |  |  |  | 4 | -58.381 | 0.558 | 125.614 | 6.158 |
| **9** | + | + | + |  | + |  |  | 7 | -59.228 | 0.597 | 126.239 | 6.783 |
| **10** | + | + | + | + |  |  |  | 7 | -57.938 | 0.596 | 126.434 | 6.978 |
| **11** | + | + | + |  |  | + |  | 7 | -56.440 | 0.595 | 126.567 | 7.111 |
| **12** | + | + | + | + | + |  |  | 8 | -58.439 | 0.605 | 127.282 | 7.826 |
| **13** | + | + |  |  |  |  |  | 5 | -59.996 | 0.561 | 127.493 | 8.037 |
| **14** | + | + |  | + |  |  |  | 6 | -59.530 | 0.572 | 128.025 | 8.569 |
| **15** | + | + | + |  | + | + |  | 8 | -57.561 | 0.599 | 128.490 | 9.034 |
| **16** | + | + | + | + |  | + |  | 8 | -56.388 | 0.595 | 129.051 | 9.595 |
| **17** | + | + | + | + | + | + |  | 9 | -56.882 | 0.605 | 129.964 | 10.508 |
| **18** | + | + | + | + | + | + | + | 10 | -55.601 | 0.604 | 132.927 | 13.471 |

Table S2 – AIC output from the ‘dredge’ function comparing the relative strength of potential models explaining the relationship between termite CTmin and climatic changes in the environment in which the termites are found.

Notes: The model parameters are in columns two through eight, and the parameters included in each of the models (numbered in the first column) are denoted by a “+” sign, and a light grey highlight. Each individual parameter is numbered, and the interactions between these parameters are indicated by these numbers in columns five to eight. The number of degrees of freedom lost in the model (k), the log-likelihood of the model, the R^2^ of the model, the AICc value, and the difference in the AICc value of the model and the model with the lowest AICc (∆AICc) are also shown. In all models, the random effect of sampling location is included. Please note, rainfall was rescalled from annual rainfall to average daily rainfall to account for differences in size with the other explanatory variable

**Fig. S1** Maps of the environmental conditions, and our sampling locations in Ghana; **a** represents mean maximum temperature; **b** represents mean minimum temperature; and **c** represents average annual rainfall. All data averaged over a thirty-year dataset (between 1970–2000) taken from WorldClim2 (Fick and Hijmans, 2017), of recorded and interpolated environmental data. Black points denote a representative location for our sampling locations. The most northerly two points are our savanna sampling sites (Mole North, the more northerly of the two, and Mole South, the more southerly); the central two points are the FoRIG Campus (more westerly of the two) and Bobiri Forest Reserve (more easterly); and the two most southerly points are Kakum Farmland (more westerly point of the two) and Kakum National Park (more easterly).

**c**

**b**

**a**


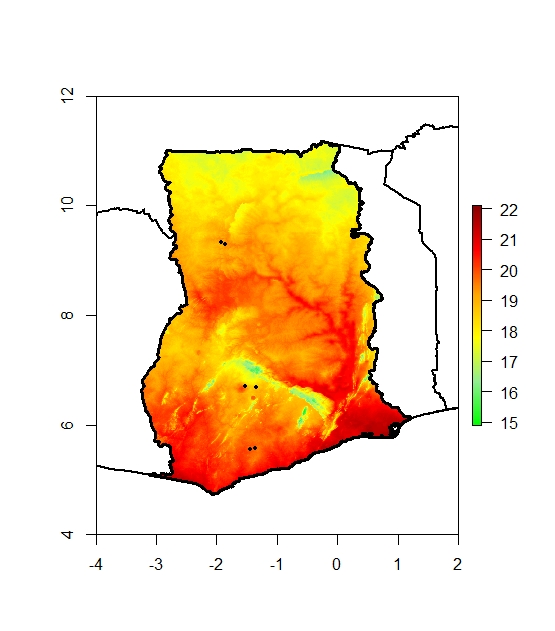

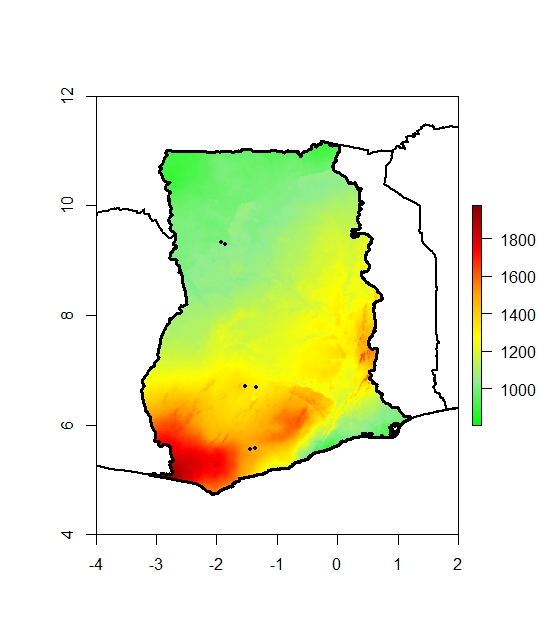

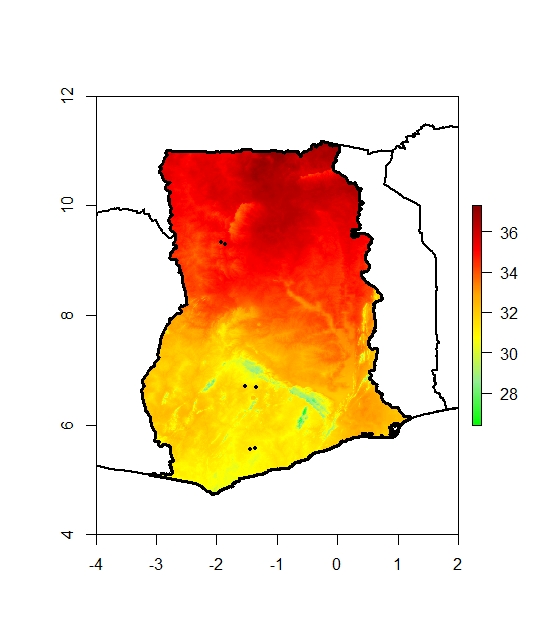

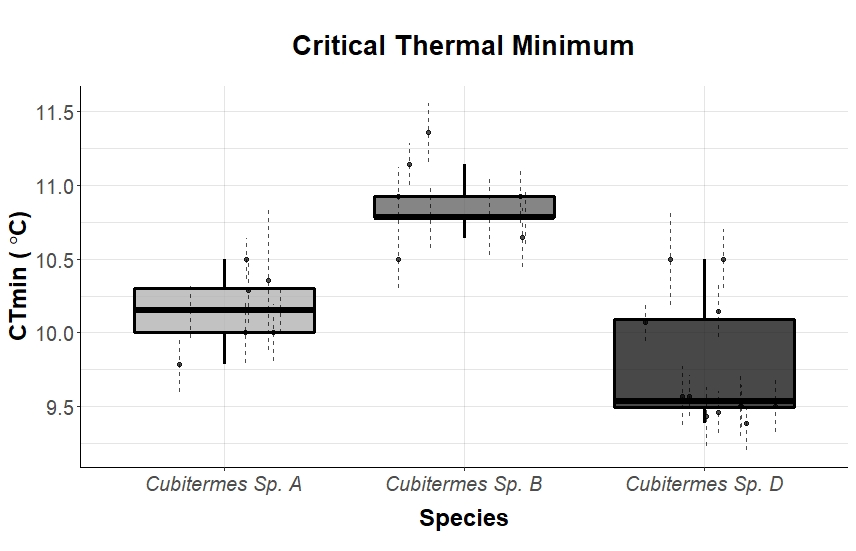


**a**

**a**

**b**

**Fig. S2** The three *Cubitermes* species sampled and their CT_min_ distributions. Species A and Species B were both sampled from forest environments, whereas Species D was sampled from the savanna environment. The thick central line of the boxplots represent the species median, and the boxes represent the interquartile range. The whiskers represent the upper and lower adjacent values. Each point represents a unique colony, with the point itself representing the colony mean, with the dotted lines denoting the standard error around this average. Letters denote the categories that the Tukey’s HSD sorted the three species into, with differences in letters representing a significant difference in CT_min_. At the time of the genetic analysis, the *Cubitermes* genus had not been split into multiple different genera, so all species here are considered *Cubitermes sensu lato*.


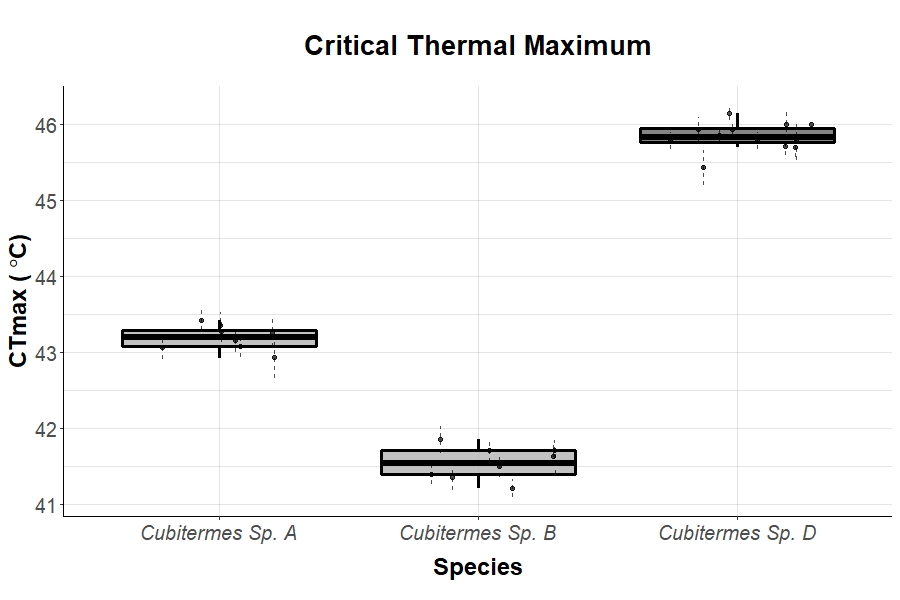


**a**

**b**

**c**

**Fig. S3** The three *Cubitermes* species sampled and their CT_max_ distributions. Species A and Species B were both sampled from forest environments, whereas Species D was sampled from the savanna environment. The thick central line of the boxplots represent the species median, and the boxes represent the interquartile range. The whiskers represent the upper and lower adjacent values. Each point represents a unique colony, with the point itself representing the colony mean, with the dotted lines denoting the standard error around this average. Letters denote the categories that the Tukey’s HSD sorted the three species into, with differences in letters representing a significant difference in CT_max_. At the time of the genetic analysis, the *Cubitermes* genus had not been split into multiple different genera, so all species here are considered *Cubitermes sensu lato.*


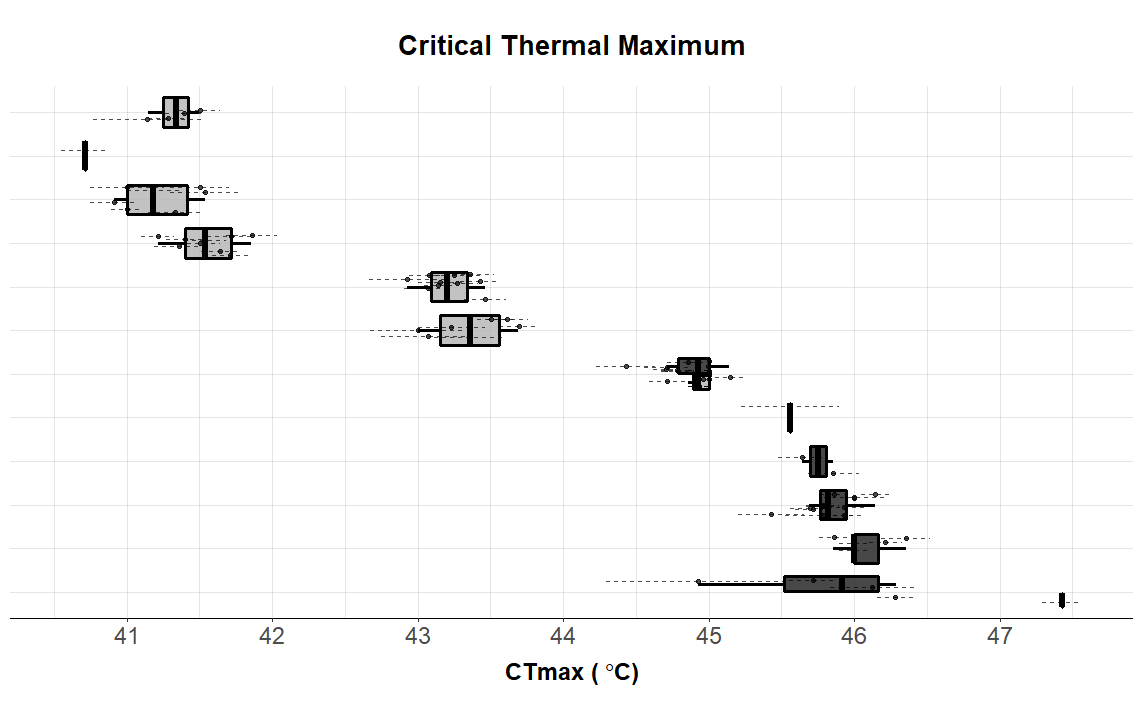

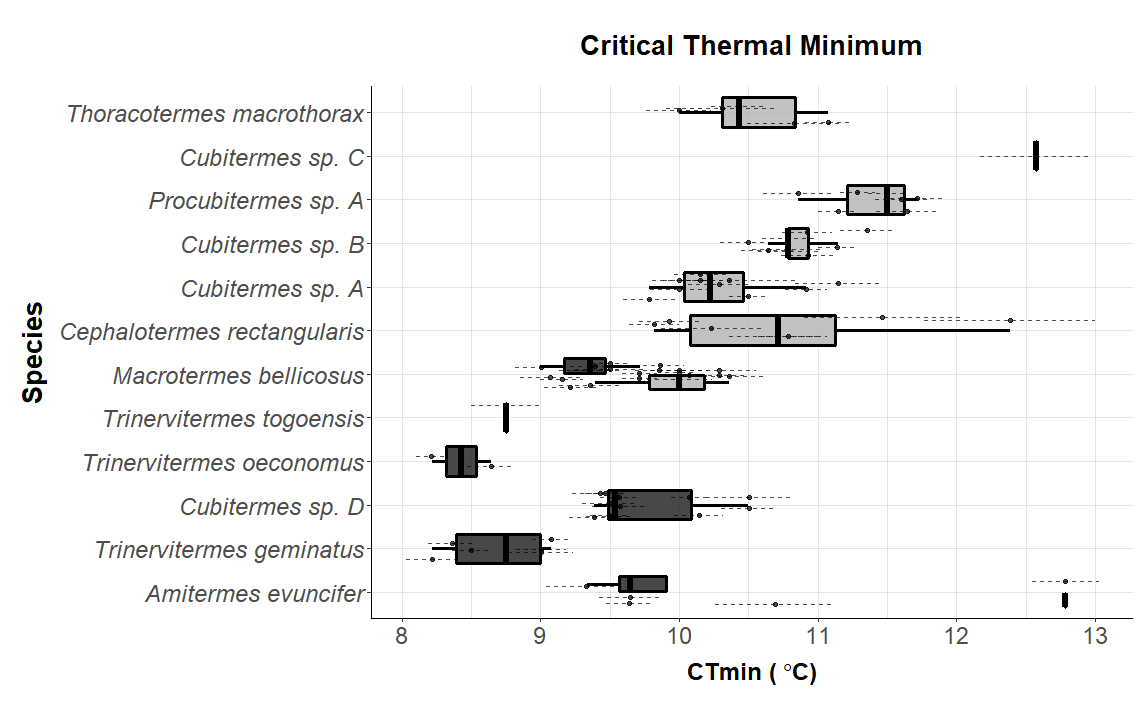


Fig. S4 The relationship between the thermal limits of all 12 termite species sampled, and the habitat they were sampled from. This some species not used in the final analyses; Trinervitermes oeconomus, Trinervitermes togoensis, Cubitermes species C, and Amitermes evuncifer. These species were removed from the final analyses primarily due to lack of colony replicates. Species are ordered from lowest CT_max_ (top) to highest CT_max_ (bottom). Colour of the boxplot denotes the location the termites were sampled, dark-grey from savanna and light-grey from forest. The points represent the estimated thermal limit of a unique termite colony, and the dotted lines represent the standard error around the colony average. Central thick line of the boxplot represents the species median thermal limit, the box represents the interquartile range, and the whiskers the upper and lower adjacent values. Note that a number of species were only sampled once, thus are not represented by a box. Cubitermes species C was sampled from a tropical forest, as was a single colony of Amitermes evuncifer. Trinervitermes togoensis was sampled from a savanna.
